# Immune cell targeting nanoparticles: a review

**DOI:** 10.1186/s40824-021-00246-2

**Published:** 2021-12-20

**Authors:** Na Kyeong Lee, Se-Na Kim, Chun Gwon Park

**Affiliations:** 1 Department of Intelligent Precision Healthcare Convergence, Sungkyunkwan University, Suwon, Gyeonggi 16419 Republic of Korea; 2 Institute of Medical & Biological Engineering, Medical Research Center, Seoul National University, Seoul, 03080 Republic of Korea; 3 Department of Biomedical Engineering, SKKU Institute for Convergence, Sungkyunkwan University (SKKU), Suwon, Gyeonggi 16419 Republic of Korea; 4 Biomedical Institute for Convergence at SKKU (BICS), Sungkyunkwan University, Suwon, Gyeonggi 16419 Republic of Korea; 5 Center for Neuroscience Imaging Research, Institute for Basic Science (IBS), Suwon, Gyeonggi 16419 Republic of Korea

**Keywords:** Target drug delivery, Nanoparticles, Surface modification, Immune cells, Active targeting, Target ligand, Enzymatic response

## Abstract

Immune cells are attractive targets for therapy as they are direct participants in a variety of diseases. Delivering a therapeutic agent only to cells that act on a disease by distinguishing them from other cells has the advantage of concentrating the therapeutic effect and lowering systemic side effects. Distinguishing each immune cell from other immune cells to deliver substances, including drugs and genes, can be achieved using nanotechnology. And also nanoparticles can ensure in vivo stability and sustained drug release. In addition, there is an ease of surface modification, which is an important characteristic that can be utilized in targeted drug delivery systems. This characteristic allows us to utilize various properties that are specifically expressed in each immune cell. A number of studies have delivered various substances specifically to immune cells through surface engineering with active target ligands that can target each immune cell and enzyme-responsive coating, and demonstrated high therapeutic effects compared to conventional treatments. Progress in research on target delivery has been suggested to be a breakthrough for the treatments of various diseases, including cancer treatment.

## Introduction

Immune cells are a major part of the immune system that develop from stem cells in the bone marrow and differentiate into granulocytes, macrophages, dendritic cells (DCs), T cells, B cells, natural killer cells (NK cells), etc. [1]. They regulate immunity in response to changes in the body caused by external and internal environments and protect the host from invading pathogens, foreign substances, and malignancies [2]. The disturbed immune system causes either suppression or overstimulation of immune cells and affects the onset and exacerbation of disease [3–5]. For example, PD-L1 and PD-L2 expressed on tumor cells are ligands of key immune checkpoint receptors of activated T cells and mediate immunosuppression of T cells, making them a major target for anticancer immunotherapy [6]. In contrast, plaques that induce atherosclerosis, the main cause of cardiovascular diseases, are caused by excessive monocyte recruitment and cholesterol efflux; hence, a monocyte-modulating strategy is essential for treatment [7]. Therefore, it is important that all components of therapeutic agents, including chemical and biopharmaceutical compounds, should be properly delivered to the targeted immune cells, since the immune cells targeted for treatment and their functional regulation strategies are different for each disease.

**Scheme 1:**
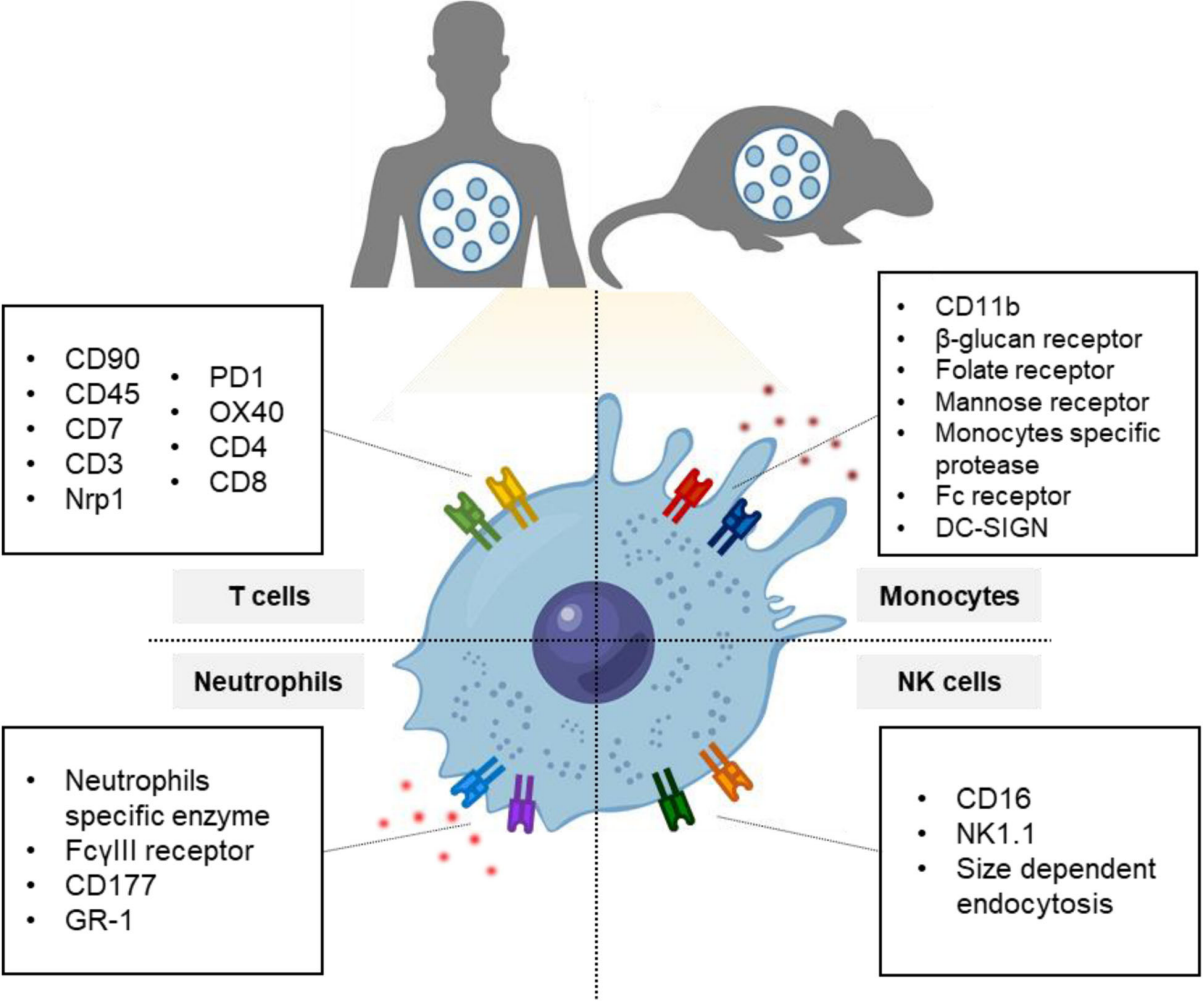
Schematic representation of cell surface markers and properties of each immune cells used as target delivery strategies.

Advances in nanotechnology have driven growth in the biological and medical fields [8]. Various nanoparticles (NPs) such as liposome, polymeric NPs, and inorganic NPs offer advantages in drug delivery applications, including ease of surface modification, improvement of in vitro and in vivo drug stability, and therapeutic efficacy [9]. Engineered NPs can be designed to control drug release and target or avoid specific interactions with various cells to focus drug delivery to target sites and avoid enzymatic degradation [10–12]. In this regard, the use of NPs makes it possible to deliver drugs to specific immune cells, thereby increasing the therapeutic effect by concentrating the drug on the site of action and lowering systemic side effects [13, 14]. Therefore, many studies have been conducted on the use of NPs for target delivery; moreover, applications in immune cell targeting are being developed. In this review, we present an overview of NPs utilizing immune cells, including targeting immune cells and modulating the activity of specific immune cells (Scheme 1).

## Immune cells targeting nanoparticles

### T cells targeting nanoparticles

T cells are involved in lymphocytes and play a central role in the adaptive immune response [15]. One of the functions of T cells is immune-mediated cell death. There are two major subtypes of T cells: CD8+ and CD4+ T cells [16]. CD8+ T cells, also known as killer T cells, directly kill virus-infected cells and cancer cells [17]. In contrast, CD4+ T cells play an indirect role in the death of infected cells by determining whether and how the immune system responds to a perceived threat or by regulating other immune cells through cytokines [18]. As such, T cells are not only involved in the response to various diseases, including cancer, but also have several cell markers on their surface, so there are several target strategies that can be applied to various diseases (Table 1).

**Fig. 1:**
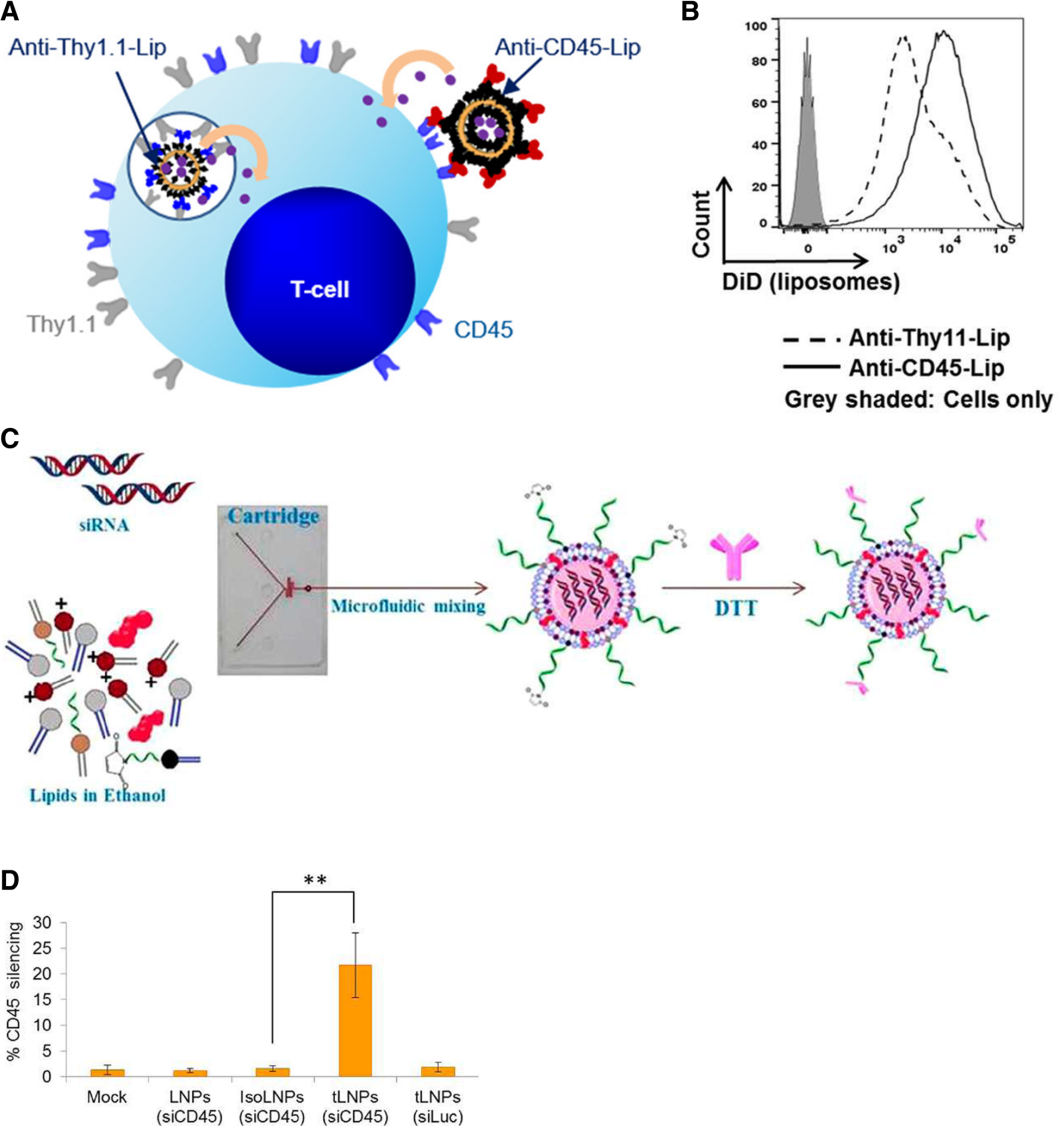
**A** Schematic illustration of anti-Thy 1.1 or anti-CD45 conjugated liposome for T cell targeting. **B** Mean fluorescence of pmel-1 Thy1.1+ T-cells after co-incubation with anti-CD45-liposome or anti-Thy1.1-liposome analyzed by flow cytometry. **C** Schematic illustration of the process in which anti-CD4 Ab functionalized lipid NPs were prepared for siRNA delivery to T cell. **D** Percentage of CD45 silencing CD4+ T cells in blood 5 days after intravenous injection into mice with saline (mock), LNPs (siCD45) tLNPs (siCD45), isoLNPs (siCD45), or tLNPs (siLuc); data represent mean ± SD, *n* = 5, **p < 0.005. **A, B** Reprinted from [19], copyright permission by American Chemical Society 2017. **C, D** Reprinted from [20], copyright permission by American Chemical Society 2015

Among the various T cell surface markers, Zheng et al. used an internalizing receptor (CD90 or Thy 1.1) and a non-internalizing receptor (CD45) to prepare transforming growth factor-β (TGF-β) inhibitor-entrapped liposomes and compared both liposomes (Fig. 1A) [19]. This approach overcomes the limitations of adoptive cell therapy (ACT) because of the loss of ACT T cell effector function by tumor-secreted immunosuppressive cytokine transforming growth factor-β (TGF-β). All liposomes promoted granzyme expression in T cells compared with the systemic administration of TGF-β (Fig. 1B). However, among liposome-preloaded T cells ex vivo, CD45 targeting liposome led to the greatest accumulation of T cells in the tumor site, and when directly targeting ACT T cells in vivo, CD90 targeting liposome showed the best tumor therapeutic effect. Because CD45 is the receptor that is expressed on the surface of all hematopoietic cells and their precursors, in vivo targeting of ACT T cells using anti-CD45 would be complicated.

CD7 is a receptor found in mature human T cells [21]. Lee et al. studied chitosan NPs conjugated with a CD7-specific single chain antibody (scFvCD7 Ab) by carbodiimide chemical to improve the intracellular delivery efficiency of siRNA. Another T cell targeting receptor is CD3, and biodegradable poly(β-amino ester)-based NPs coupled with f (ab’)2 fragments of an anti-CD3e Ab successfully delivered genes encoding disease-specific chimeric antigen receptors (CARs) in T cells [22]. Similarly, anti-CD3 Ab-decorated protein NPs composed of helix-rich bacterial inclusion bodies (IBs) successfully captured T cells [23]. In addition, these NPs enabled the redirecting of T cells specifically to cancer cells through dual decoration of an Ab targeting epidermal growth factor receptor (EGFR) expressed by tumor cells. Another dual-targeting NP spatiotemporally co-delivered anti-PD1 and OX40 Abs to T cells to improve T cell activation [24]. It was confirmed that these dual immunotherapy NPs showed greater anticancer effects than free administration of Abs.

In addition, studies have targeted specific subtypes of T cells. Kovochich et al. developed an anti-human CD4 Ab conjugated liposome to deliver the HIV activator protein kinase C activator bryostatin-2 (Bry) [25]. As an antiretroviral therapy, Bry makes virus-infected cells a target for the immune system by activating the latently infected cells to induce virus production. According to the fluorescent microscopy image, CD4 T cells targeting liposomes preferentially attached to CD4 T cells compared to isotype Ab conjugated liposomes. In addition, since the site of action for Bry is the intracellular membrane, target delivery liposomes activated latent HIV more efficiently than Bry alone at all doses. Human CD4+ T cells and murine CD4+ T cells can be targeted via surface-functionalized lipid NPs with anti-CD4 monoclonal Abs, which increase the delivery efficiency of small interfering RNA (siRNA) and modulate T cell functions (Fig. 1C, D) [20]. Neuropilin-1 (Nrp1), a cell surface transmembrane glycoprotein, is exclusively expressed in regulatory T (Treg) [26], a type of CD4 + T cell and immunosuppressive cell, so Nrp1 binding peptides can be used as a target strategy [27]. CD8+ T cells, which are effector T cells, can be targeted through an anti-CD8a Ab [28]. Yang et al. conjugated anti-mouse CD8a Abs to small cell membrane-penetrating amphiphilic gold nanoparticles and confirmed their ability to target CD8 + T cells in mice. In addition, the expression of transferrin receptors is increased in activated T cells (ATCs), which can be used as a targeting strategy for ATCs [29]. Xie et al. produced a polyplex of transferrin-polyethylenimine (PEI) and siRNA as a targeted delivery system to deliver silence inflammation-related genes in ATCs, and confirmed its potential as a therapeutic agent for asthma.

### Monocyte targeting nanoparticles

**Fig. 2:**
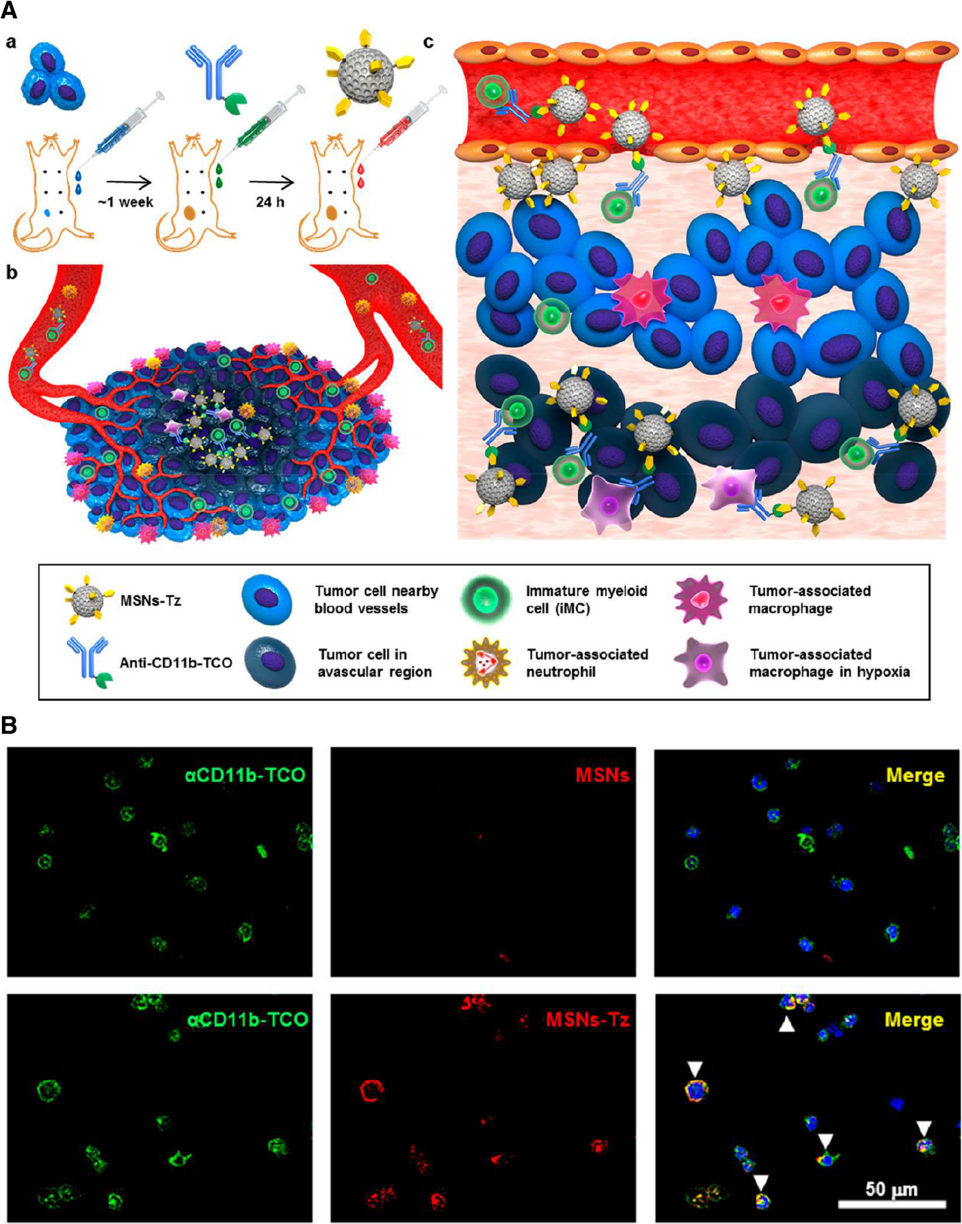
**A** CD11b + monocytes in the tumor microenvironment and blood vessels were labeled by anti-CD11b-TCO and then tagged with MSNs-Tz by click chemistry between TCO and Tz. The labeled CD11b + monocytes carrying the drug loaded in MSNs penetrated blood vessel toward the tumor microenvironment. **B** Bottom row confocal microscopy images show targeting of MSNs-Tz (visualized in red) to anti-CD11b-TCO (visulaized in green) on the surface of bone marrow-derived cells. As control group, MSN lacking Tz is in the top row of image. **A, B** Reprinted from [31], copyright permission by American Chemical Society 2019

Monocytes are a type of leukocyte that can differentiate into macrophages and conventional DCs. In addition, various cellular markers that can be used for target delivery have been reported (Table 2). The These cells are distinguished from other cells by the expression of CD11b, a monocyte cell marker [30]. Therefore, using Ab to capture CD11b is a promising strategy for targeting monocytes. Lee et al. developed a click reaction-assisted immune cell targeting for delivering drugs into deep tumor regions using trans-cyclooctene (TCO)-modified anti-CD11b Abs and mesoporous silica nanoparticles functionalized with tetrazines (MSNs-Tz) (Fig. 2A, B) [31]. Because the click chemistry between TCO and TZ is faster than other click reactions, sequential injection of TCO-modified anti-CD11b Ab and MSNs-Tz led to their conjugation through fast and catalyst-free reactions in vivo. This conjugated drug delivery system successfully targeted monocytes and delivered drugs to the deep tumor site by monocyte hitchhiking.

**Fig. 3:**
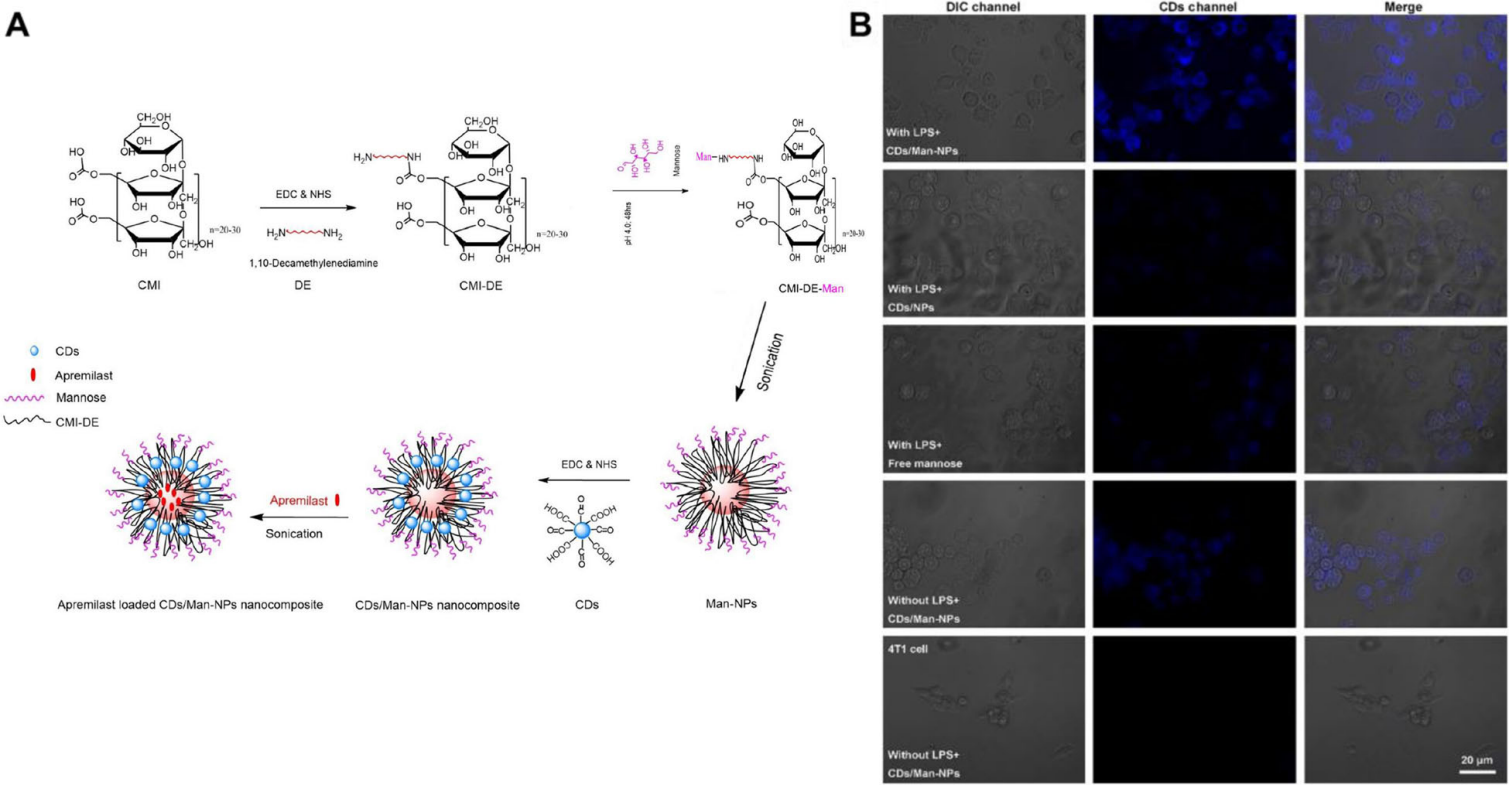
**A** Synthetic scheme of the drug loaded CDs/Man-NPs nanocomposite. **B** Cell uptakes of activated macrophages, untreated macrophages and 4 T1 cell were assessed by confocal laser scanning microscopy. Each cells were treated with CDs/Man-NP and unmodified CDs/NPs, respectively. The differential interference contrast microscopy of cells, NPs (blue) and overlays of the two images. Scale bar = 20 μm. **A, B** Reproduced from [40], copyright permission by Elsevier 2020

Some studies have explored targeting systems specific to macrophages among all types of monocytes using the receptors on macrophages. As phagocytic cells, macrophages have β-glucan receptors for the recognition and phagocytosis of fungal, plant, and bacterial β-glucan-linked carbohydrates [32, 33]. Ren et al. reported that RAW 264.7 cells efficiently endocytosed yeast cell wall-coated poly (lactide-co-glycolide) (PLGA) NPs in vitro [34]. Moreover, the folate (FA) receptor on the surface of monocytes can be used to target monocytes, especially activated macrophages [35]. In a mouse model of ulcerative colitis and atherosclerosis, the designed poly (ethylene glycol)-coated, acetyl-capped, folate-functionalized poly (amidoamine) (PAMAM) dendrimer was able to selectively capture a folate-receptor-expressing macrophage cell line in vitro and accumulate specifically at inflammation sites in vivo without any discernible cytotoxicity [36]. In addition, silver NPs that induced M1 macrophage apoptosis and facilitated M2 macrophage polarization were decorated with FA for the treatment of rheumatoid arthritis [37]. In addition to the FA receptor, the mannose receptor is a well-characterized membrane receptor on the surface of macrophages [38, 39]. Mannosylated decamethylenediamine-grafted carboxymethyl inulin amphiphilic acid NPs with carbon dots (Fig. 3) [40] and PAMAM dendrimers have been developed for specific delivery to macrophages [41].

In addition to targeting macrophage-specific receptors, there is also a dual targeting method that uses proteases and enzymes secreted by macrophages. Liang et al. designed a dual functionalized nanoparticle platform by conjugating legumain protease degradable poly polyethylene glycol (PEG) and tuftsin to an NP surface for selective targeted delivery to tumor-associated macrophages (TAMs) [42]. Legumain protease is overexpressed in TAMs [43] and tuftsin is a tetrapeptide located in the Fc domain of the Abs [44]. Similarly, Deng et al. reported macrophage-specific enzyme-responsive PLGA NPs composed of matrix metalloprotease 9 (MMP-9) cleavable PEG and surface modification with arginylglycylaspartic acid (RGD) [45]. These PLGA NPs successfully targeted inflammatory macrophages derived from patients with rheumatoid arthritis via an RGD-αvβ3 integrin interaction after PEG cleavage by MMP-9.

In addition to Abs, peptides can also be applied to macrophage-targeting NPs. Han et al. reported that PLGA NPs with a designated M2-like macrophage binding peptide (M2pep, YEQDPWGVKWWY) and scavenger receptor B type 1 (SR-B1) targeting peptide (α-peptide) bind with greater specificity to M2-like TAMs than to other leukocytes [46].

In DCs, the mannose receptor is a member of the dendritic cell-specific intercellular adhesion molecule 3-grabbing non-integrin (DC-SIGN), including DC-SIGN (CD209), the liver and lymphatic endothelium homologue of DC-SIGN (L-SIGN), and langerin (CD207) [47]. A novel DC-targeting lipid NP containing mannose-mimicking di-shikimoyl- and guanidine head groups and two n-hexadecyl hydrophobic tails showed significantly enhanced uptake by CD11c + DCs compared with control lipid NPs [48]. The electrostatic complex of these lipid NPs and DNA vaccine encoding melanoma tumor-associated antigens also provided notable anti-melanoma immunity with a memory response in up to 80% of vaccinated mice. Similarly, mannosylated gelatin NPs loaded with inactivated porcine reproductive and respiratory syndrome virus (PRRSV) led to the maturation of DCs to induce T cell-mediated immunity [49]. The complex of mannose coagulated ovalbumin (OVA) and PEI via electrostatic reaction contributed to cancer immunotherapy by accelerating endosomal escape and enhancing MHC-I antigen presentation in DCs [50]. Similar to Mannose-functionalized NPs, dextran-functionalized NPs also have a high affinity to DC-SIGN [51]. In addition to using DC-SIGN targeting Abs, Stead et al. created an in vivo DC targeting method by further coupling an anti-CD11b Ab to porous silicon NPs [52].

### NK cells targeting nanoparticles

**Fig. 4:**
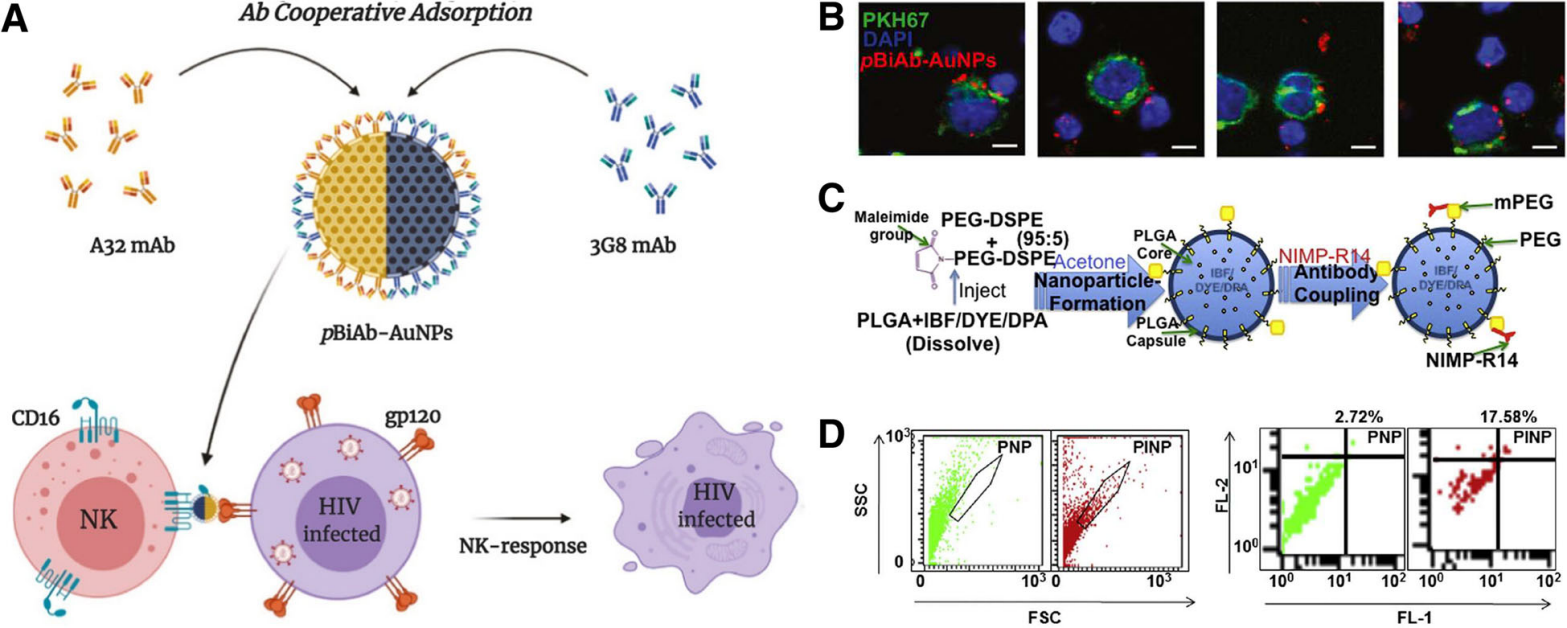
**A** Schematic illustration of AuNPs for the specific enhancement of the immune response of NK cells towards HIV-infected T cells. **B** Representative confocal image of HIV-expressing cells coated with HIV gp120 recombinant protein (stained with PKH67 in green, and DAPI nuclei staining in blue) and NK cells (DAPI nuclei staining). Both cell types were co-cultured at 1:1 ratio for 20 min in the presence of the pBiAb-AuNPs (visualized in red). Scale bar = 5 μm. **C** Schematic synthesis process of the neutrophil-targeted Ab conjugated and drug or fluorescent-dye loaded PLGA-PEG NPs. **D** The forward scatter (FSC), side scatter (SSC) plots of PEGylated NPs (PNPs) or NIMP-R-14 Ab conjugated-PNPs (PINP) were analyzed by flow cytometer. The shift in FSC/SSC for PINPs indicated change in particle size due to NIMP-R-14 Ab conjugation (Left). The NIMP R-14 (FL2) positive neutrophils were isolated from PNP or PINP treated mice and analyzed for PINP delivery (FL1) to neutrophils. Flow data shows significantly higher delivery by PINP as compared to the PNP as negative control (Right). **A, B** Reproduced from [53], copyright permission by Elsevier 2020. **C, D** Reprinted from [54], copyright permission by Elsevier 2016

NK cells are a type of cytotoxic lymphocyte included in the innate immune system and can be targeted using various characteristics (**Table 3**).CD16, also known as FcγRIII, is found on the surface of NK cells and mediates ab-dependent cellular cytotoxicity. It is expressed in both humans and mice, and since it is an NK cell activation molecule, it can induce NK cell activity at the same time as the target. Astorga-Gamaza et al. developed a bispecific gold NP, double-conjugated with two Abs targeting HIVgp120 on the membrane of HIV-infected cells (anti-HIVgp120) and NK cells (anti-human CD16) (Fig. 4A) [53]. Using a linker-free conjugation method that promotes the ordered distribution and separation of Abs, the cooperatively adsorbed Abs were capable of recognizing their antigen and significantly enhanced cell-to-cell contact between HIV-expressing cells and NK cells (Fig. 4B). CD16 was also used to target mouse NK cells. Au et al. conjugated anti-mouse CD16 Abs to PEG-PLGA NPs [55]. Additionally, they conjugated anti-mouse 4-1BB and anti-human EGFR Abs. As a result, these tri-specific PEG-PLGA NPs demonstrated effective NK cell activation by the spatiotemporal co-activation of CD16 and 4-1BB stimulatory molecules on NK cells and promoted NK cell recruitment to EGFR-positive tumor cells. In a mouse, NK1.1 can be used for targeting mouse NK cells. Chandrasekaran et al. studied liposomes decorated with tumor necrosis factor-α related apoptosis-inducing ligand (TRAIL), which initiates apoptosis by interacting with death receptors on cancer cells and anti-mouse NK1.1 Ab on its surface [56]. These NK cell-targeted liposomes successfully delivered TRAIL to NK cells and enhanced the NK cell-mediated anti-tumor immune response.

In addition to activating the targeting ligand, size is also an important factor in NK cell-targeting NPs. Adjei et al. showed the effect of the size of polystyrene NPs on uptake by NK cells [57]. They incubated NK92 cells with fluorescently labeled polystyrene NPs that were 20, 30, 40, and 100 nm in diameter. After incubation for up to 24 h, confocal images showed that smaller NPs were taken up uniformly in a shorter time. These findings may be indicative of the endocytic mechanisms utilized by NK cells for particle uptake. In general, particles less than 100 nm in diameter predominantly utilize clathrin-mediated pathways of endocytosis, while those larger than 100 nm utilize caveolae-mediated pathways and micropinocytosis [58]. Identification of these mechanisms of NPs uptake will be necessary for the development and optimization of NPs targeting NK cells.

### Neutrophil targeting nanoparticles

Neutrophils are a type of phagocyte found in the blood and are among the first responders in the initial phase of inflammation caused by bacterial infection, changes in the environment, and cancer cells [59, 60]. After migration to the inflammation site, neutrophils ingest and kill microbes or release antimicrobial proteins in granules by a process called degranulation. However, uncontrolled activity of neutrophils can lead to disruption of tissue showing symptoms corresponding to acute injury due to excessive secretion of granules containing protease and digestion enzymes [61]. In other words, it is possible to design a target delivery strategy by utilizing these points along with cell surface markers (Table 4). The most prominent example is intractable lung or airway inflammation characterized by sustained polymorphonuclear neutrophil recruitment to the airways [62]. Therefore, pulmonary drug delivery can achieve high target drug delivery efficiency by using an excess of proteases and digestive enzymes specifically secreted by neutrophils. Mejías et al. constructed a self-regulated nanoparticle-in-microgel system (N in M), in which extracellular elastase degranulated by inflammatory neutrophils dissociates microgel to release nanoparticle-loaded Nexinhib20, a potent neutrophil degranulation inhibitor [63]. Successful in vivo delivery of Nexinhib20 by N in M to the airways and neutrophils dampened neutrophil recruitment and degranulation, leading to resolution of the inflammatory response. A similar study was conducted for myeloperoxidase (MPO)-targeting NPs, which is a targeting strategy using another neutrophil-specific enzyme [64]. These NPs consisted of PEG-PLGA and synthesized MPO targeting ligand, 5-hydroxytryptamine (5-HT), and targeted neutrophils in the tumor, which could enhance retention and achieve sustained release of the drug, increasing anti-cancer therapeutic and anti-metastasis efficiency.

The material of NPs selected as drug carriers is an important strategic element for neutrophil targeting. Wang et al. showed that albumin nanoparticles are largely internalized by neutrophils [65]. These albumin NPs do not contain any targeting ligand, rather they rely only on endocytosis mediated by the FcγIII receptor and unknown receptors. However, there is a method that uses a target ligand that specifically binds to neutrophils. CD177 is a glycosylphosphatidylinositol (GPI)-anchored glycoprotein expressed exclusively by neutrophils in the blood [66]. The function of CD177 has not been clearly clarified, but it forms a complex with neutrophil proteinase 3 and binds to platelet endothelial cell adhesion molecule 1 (PECAM-1), which enhances neutrophil transmigration [67, 68]. Crosslinking of CD177 does not activate immune responses, such as oxidative burst or degranulation, but induces internalization [69]. Although CD177 positive neutrophils are found only in 30–70% of circulating neutrophils, this neutrophil population has an advantage in transendothelial migration, making it an excellent target for reducing the accumulation of neutrophils in damaged tissues [70]. Liposomes conjugated with CD177 binding peptides, identified by Miettinen et al., were synthesized to deliver antisense oligonucleotides for the knockdown of pro-inflammatory proteins, such as the C5a receptor [71]. The success of neutrophil targeting by peptide-conjugated liposomes was confirmed by CD177-expressing Chinese hamster ovary cells, as well as human and mouse neutrophils. For targeting murine neutrophil, GPI-anchored protein Ly-6G is a good marker of peripheral neutrophils. Together with Ly-6C, this protein is a component of the myeloid differentiation antigen Gr-1 and NIMP-R14 Ab, which are highly specific for murine Ly-6G and Ly-6C [72, 73]. Vij et al. conjugated this Ab to PEGylated PLGA NPs via covalent bonding between the thiol of Ab and maleimide of PEG (Fig. 4C). This immune-conjugated PLGA selectively delivered drugs to murine neutrophils (Fig. 4D) [54].

## Conclusions

Advances in nanotechnology have had great impacts on the medical field, and various new treatments through drug delivery have been developed. Drug delivery using NPs has advantages over conventional drug delivery because it enables guaranteed in vivo drug stability, regulated drug release, and targeted drug delivery. Various materials constituting the NPs and the fact that they can be easily modified are characteristics that can overcome the limitations of existing treatments. In particular, circulating immune cells in the body, which are attractive targets for drug delivery because they directly participate in various diseases, are effectively and easily targeted using NPs. A number of studies have developed targeted drug delivery systems using surface markers specifically expressed on each immune cell, or using enzymes or proteases secreted by the cell. Thus, it was confirmed that the substances, including drugs and genes, were effectively delivered only to the site of application, thereby concentrating the therapeutic effect and lowering the systemic side effects.

In the future, further studies on the markers specifically expressed on the surfaces of each immune cell and the mechanism of cellular uptake can lead to the development of targeted drug delivery systems. Through this, it is expected that it will be possible to develop targeted drug delivery systems that can be applied to various diseases and improve treatment effects and patient outcomes compared to existing drug delivery methods.

## Data Availability

Not applicable.

## Abbreviations

DCs: Dendritic cells
NK cells: Natural killer cells
Treg: Regulatory T
NPs: Nanoparticles
siRNA: Small interfering RNA
Antibody: Ab
PLGA: Poly (lactide-co-glycolide)
PEG: Polyethylene glycol
TAMs: Tumor associate macrophages
Nrp1: Neuropilin-1
EGFR: Epidermal growth factor receptor
TGF-β: Transforming growth factor-β
PAMAM: Poly (amidoamine)
PEI: polyethylenimine
M2pep: M2-like macrophage binding peptide
OVA: ovalbumin
α-peptide: Scavenger Receptor B type 1 targeting peptide
TRAIL: Tumor necrosis factor-α related apoptosis inducing ligand
GPI: glycosylphosphatidylinositol (GPI)
